# Evaluation of *NDRG2 *gene expression in primary papillary thyroid carcinoma and in metastases of this neoplasm to regional lymph nodes

**DOI:** 10.1186/1756-6614-3-6

**Published:** 2010-08-30

**Authors:** Anna Mordalska, Joanna Latek, Tomasz Ferenc, Lech Pomorski, Elżbieta Gałecka, Arkadiusz Zygmunt, Andrzej Lewiński

**Affiliations:** 1 Department of Biology and Medical Genetics, Medical University of Lodz, Lodz, Poland; 2 Department of Endocrinology and Metabolic Diseases, Polish Mother's Memorial Hospital - Research Institute, Lodz, Poland; 3 Department of General and Oncological Surgery, Medical University of Lodz, Zgierz, Poland; 4 Department of Endocrinology and Metabolic Diseases, Medical University of Lodz, Lodz, Poland

## Abstract

**Background:**

At present, researchers' attention has been concentrating on *NDRG2 *(*N-Myc *downstream-regulated gene 2) as a new gene candidate in the development and progression of papillary thyroid carcinoma (PTC). *NDRG2*, together with *NDRG1*, *NDRG3 *and *NDRG4 *are members of the *NDRG *family, a new class of genes, inhibited by *N-Myc *oncogene.

**Aim:**

The aim of our study was to evaluate *NDRG2 *mRNA expression in the primary PTC and its metastases to regional lymph nodes.

**Materials and methods:**

Postoperative tissue and macroscopically changed lymph nodes of sixteen (16) patients with PTC constituted the studied material. In this group, metastases of the cancer to regional lymph nodes were confirmed histopathologically in 8 cases. Quantitative evaluation of *NDRG2 *mRNA expression was performed by the real-time polymerase chain reaction (real-time PCR) method.

**Results:**

The mean values of *NDRG2 *mRNA expression in the primary tumour tissues were statistically significantly lower vs. the levels of *NDRG2 *mRNA expression in macroscopically unchanged thyroid tissue (p < 0.0001). A comparison of the mean *NDRG2 *mRNA expression of primary tumours and that of their metastases to regional lymph nodes did not demonstrate any statistical differences (p > 0.05). A positive correlation was observed between *NDRG2 *mRNA expression in primary tumour cells and in the cancer metastases to lymph nodes (Rs = 0.7857; p < 0.05). Factors, such as age, sex, tumour stage in TNM system, were of no significance for *NDRG2 *mRNA expression level (p > 0.1).

**Conclusion:**

The results of our study demonstrated decreased *NDRG2 *mRNA expression levels in PTC, when compared to macroscopically unchanged thyroid tissue, which may point to the potential role of *NDRG2 *in the development and progression of cancer in question.

## Introduction

Molecular disorders, associated with papillary thyroid carcinoma (PTC) development and progression, have for many years been and still are the subject of extensive and intensive research [1-4].

Currently, researchers' attention has been concentrating on *NDRG2 *(*N-Myc *downstream-regulated gene 2), a new candidate gene, taking part in the development and progress of PTC. Deng et al. [5] were the first to have identified the human *NDRG2 *(*N-Myc *downstream-regulated gene 2). The to-date's data indicate a significant role of the *NDRG *family in the proliferation and differentiation of cells. *NDRG2 *is localised on 14q11.2 chromosome and encodes a protein of 41 kDa weight [6]. Studies of *NDRG2 *human gene expression at mRNA level have demonstrated the presence of a transcript of this gene in cells of the brain, the liver and the kidneys. The highest expression levels of the gene have been found in salivary glands, muscle and nervous cells, while *NDRG2 *mRNA was almost undetectable in the thymus, the bone marrow and leukocytes of peripheral blood. Such patterns of expression suggest a reverse correlation between the expression level of the gene and the cell proliferation rate [7]. A reduced *NDRG2 *gene expression has also been observed in a number of human neoplasms, which suggests its role as a suppressor gene of neoplasm [5,8-10].

It has been demonstrated that *NDRG2 *gene, in combination with *TP53 *gene, play a significant role in the process of apoptosis in response to DNA lesion, and *NDRG2 *is an inhibitor of cellular proliferation, regardless of the status of *TP53 *in cell [11]. The human *NDRG2 *is inhibited by *Myc *oncogene. The to-date's research has indicated that an increased expression of *Myc *oncogene is associated with decreased *NDRG2 *gene expression in many neoplasms [12]. Studies, performed on neoplastic cell lines, have demonstrated that methylation of *NDRG2 *gene promoter and point mutations within the promoter in question cause a significant reduction of *NDRG2 *gene activity [8].

Up to date, only one study concerning the *NDRG2 *gene expression in papillary thyroid carcinoma has been published in the medical literature [13].

The goal of our study was an evaluation of mRNA expression of *NDRG2 *gene in primary PTC and in its metastases to regional lymph nodes.

## Materials and methods

The procedures, as used in the study, had been approved by the Ethical Committee of the Medical University of Lodz, Poland.

Postoperative tissue samples and macroscopically changed lymph nodes of 16 patients with PTC constituted the study material. Out of them, neoplastic metastases to regional lymph nodes were histopathologically confirmed in 8 cases. The tumours were classified according to the World Health Organisation's Classification (TNM) [14]. Tissue samples were collected after total thyroidectomy, placed in storage buffer (RNA later RNA Stabilisation Reagent of Qiagen) (Hilden, Germany) and frozen in temperature of -70°C until isolation of total RNA.

Selected clinical-pathological data of patients: age, sex, histopathological diagnosis and TNM classification (acc. to WHO) are presented in Table 1.

**Table 1:** Selected clinical and pathological data of patients (age, gender), histopathological diagnosis, TNM classification (acc. to WHO)

No:	Age	Gender	Diagnosis	Grade of progression in TNM scale
1.	80	F	PTC	pT_1_N_0_M_0_

2.	19	F	PTC	pT_2_N_1b_M_0_

3.	51	F	PTC	pT_1_N_1_M_0_

4.	60	M	PTC	pT_1_N_1b_M_0_

5.	72	F	PTC	pT_4_N_1_M_0_

6.	55	F	PTC	pT_1_N_1_M_0_

7.	42	F	PTC	pT_1_N_0_M_0_

8.	58	F	PTC	pT_1_N_0_M_0_

9.	33	F	PTC	pT_1_N_0_M_0_

10.	50	M	PTC	pT_1_N_0_M_0_

11.	56	M	PTC	pT_4_N_0_M_0_

12.	48	M	PTC	pT_3_N_0_M_0_

13.	43	F	PTC	pT_1_N_1_M_0_

14.	43	F	PTC	pT_1_N_1_M_0_

15.	57	F	PTC	pT_1_N_x_M_x_

16.	35	F	PTC	pT_1_N_1_M_0_

### Isolation of total RNA and reverse transcription

Total RNA was extracted from tissues, using an RNeasy Midi Kit (Qiagen, Hilden, Germany), according to the manufacturer's recommendations.

RNA concentration was spectrophotometrically assessed by measuring absorbance at 260 and 280 nm.

Reverse transcription was performed in a personal Mastercycler (Eppendorf, Hamburg, Germany), using 1 μg of total RNA in the presence of oligo d(T) (50 μM) and MultiScribe™Reverse Transcriptase (50 U/μl) in a total volume of 30 μl, including also: 10 × TaqMan RT Buffer, MgCl_2 _solution (25 mM), dNTPs mixture (10 nM), RNAse Inhibitor (20 U/μl) and nuclease-free water (TaqMan Reverse) Transcriptase Reagents, Applied Biosystems, Foster City, California, USA).

The reactions were incubated for 60 minutes at 27°C, heated for 5 minutes to 95°C and then placed at 4°C.

### Analysis of relative *NDRG2 *mRNA amount by real-time PCR

An established Relative Qualification PCR assay for *NDRG2 *mRNA expression was used (in ABI PRISM 7500 Sequence Detection System, Applied Biosystems), according to the manufacturer's protocol. PCR reactions for *NDRG2 *were run with 50 ng of cDNA in a total volume of μl, using a TaqMan™Universal PCR Master Mix (Applied Biosystems, Foster City, California, USA) and the predesigned and labelled primer/probe set (Assay-on-Demand™Gene Expression assay mix Hs01045114_g1). After an initial, 2-minute incubation at 50°C to allow uracil-N-glycosylase digestion and at 95°C for 10 minutes to activate the Ampli Taq Gold™DNA polymerase, both of which are provided by the Universal PCR Master Mix, the samples were amplified through 50 bi-phasic cycles of 95°C for 15 s and of 60°C for 1 minute.

Macroscopically unchanged thyroid tissue served as a calibrator. The amplification reactions were performed in triplicate for each sample. Controls with no template cDNA were used with each assay.

The expression levels of glyceraldehyde-3-phosphate dehydrogenase gene (*GAPDH*) were measured as endogenous controls (reference gene), using the appropriate Assay-on-Demand™Gene Expression Mix (Hs99999905_m1) (Foster City, California, USA).

Both gene expressions were measured for each tumour sample in the same PCR reaction but in separate wells.

The Assay-on-Demand Gene Expression Mix consists of 20 × mix of unlabelled PCR primers (18 μM for each) and TaqMan™MGB probe (5 μM) with FAM™(6-carboxy-fluorescein) at the 5' end as a reporter dye and a non-fluorescent quencher (TAMRA, 6-carboxy-tetramethylrhodamine) at the 3' end. The two-minute, 50°C step is required for optimal AmpErase™UNG activity, when TaqMan™Universal Master Mix is used.

Fluorescence signal was measured in real-time in the extension phase of the PCR reaction and the measurement, proportional to the quantity of sample cDNA in the reaction, was as an amplification curve against the cycle number. A threshold value of fluorescence in the exponential part of the amplification curve was selected and, for each sample, the number of cycles was measured, which is needed by the signal to reach the threshold (threshold cycle, C_T_). The larger are the quantities of the starting material, the lower are C_T _values.

### Data analysis

Data analysis was performed by means of the Taq-Man SDS analysis software (Applied Biosystems).

Fluorescence emission data were determined as C_T _values for each reaction and, for each sample, triplicate C_T _values were averaged. The average C_T _value for *GAPDH *was subtracted from the average *NDRG2 *C_T _value to yield ΔC_T _value to yield ΔC_T _value. Normalisation to the reference gene (*GAPDH*) was necessary to account for sample quantity and quality variations, as well as for the variations of PCR efficiency among particular samples. The assay, described in this report, involves ΔΔC_T _value determination. This is calculated as: ΔΔC_T _= ΔC_T _test sample - ΔC_T _calibrator sample.

### Statistical analysis

A non-parametric U-Mann-Whitney's test was used to compare the level of *NDRG2 *expression (RQ value) between the cancer tissue and the unchanged tissue. The Spearman's rank correlation coefficient was applied to determine the correlation between gene expression in PTC and in the metastasis of the carcinoma to lymph nodes. An assessment of relationships between the level of *NDRG2 *expression and such parameters, as patient's age, sex, tumour stage (acc. to TNM) was performed with the Spearmen's rank correlation coefficient (age, tumour stage) and U-Mann-Whitney test (sex). The results are presented as basic statistics for RQ in particular groups. The statistical significance was set at p < 0.05.

Statistical analysis was performed by means of the Statistica (StatSoft, Windows 7.1) software package.

## Results

The clinical and pathological data regarding the cases of PTC are shown in Table 1.

### Differential expression of *NDRG2 *mRNA in primary tumours and normal thyroid tissues

**Figure 1:**
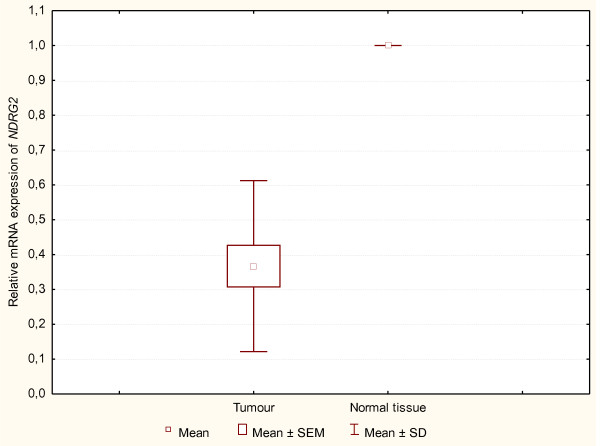
**Box and whisker chart**. The level of *NDRG2 *mRNA expression in macroscopically unchanged thyroid tissue and in PTC. The boxes represent means ± SEM. The results obtained with U-Mann-Whitney's test point to a statistically significant difference of *NDRG2 *expression in the investigated groups (p < 0.0001)

Figure 1 presents the observed mean values of *NDRG2 *mRNA expression level in PTC cells (n = 16) and in normal thyroid tissue. The reference point for the studied gene expression in PTC tissue was the value from the averaging gene expression in macroscopically unchanged thyroid tissues. The mean values of *NDRG2 *mRNA expression level in primary carcinoma (tumour) cells were significantly lower vs. the of *NDRG2 *mRNA expression level in normal thyroid tissue (p < 0.0001).

### *NDRG2 *mRNA expression in primary tumours and metastases

**Figure 2:**
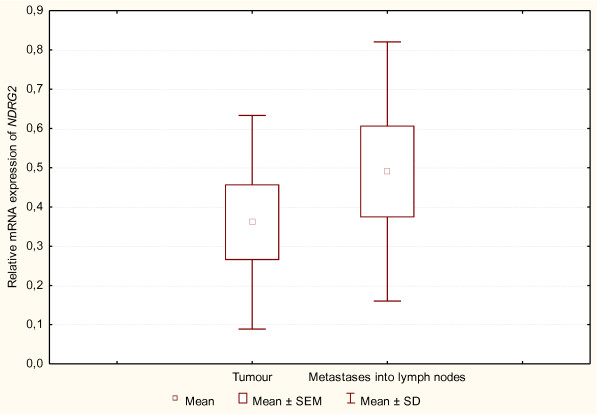
**Box and whisker chart**. The level of *NDRG2 *mRNA expression in primary tumour and in metastases to regional lymph nodes. The results obtained with Spearman's rank correlation test did not demonstrate statistically significant differences between the level of expression in the investigated groups (p > 0.05).

Figure 2 presents the recorded mean values of the *NDRG2 *mRNA expression level in primary carcinoma (tumour) cells (n = 8) and the mean values of the NDRG2 mRNA expression level in metastases of the carcinoma to regional lymph nodes (n = 8). A comparison of the mean values of those parameters between the primary carcinoma (tumour) and metastases of that carcinoma to lymph nodes did not indicate any statistically significant differences (p > 0.05). There was no statistically significant difference (p > 0.1) between the primary tumours with metastases and without metastases in regard to the NDRG2 mRNA expression.

A positive correlation was observed between the *NDRG2 *mRNA expression level in primary carcinoma (tumour) cells and the *NDRG2 *mRNA expression in the same carcinoma metastases to lymph nodes (Rs = 0.7857; p < 0.05). It means that the decrease of the *NDRG2 *mRNA expression level in primary carcinoma (tumour) was accompanied by a fall in the *NDRG2 *mRNA expression level in metastases of the carcinoma to lymph nodes.

### *NDRG2 *mRNA expression depending on tumour stage (TNM) and on patient's age and sex

**Figure 3:**
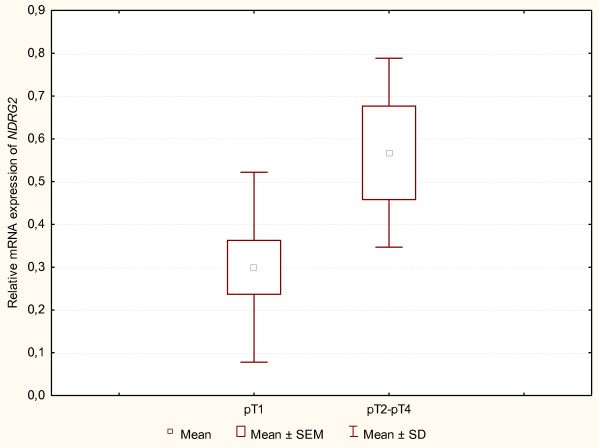
**Box and whisker chart**. The dependence of the level of *NDRG2 *mRNA expression on the tumour grade (pT, TNM scale) in two investigated PTC groups: pT1 (n = 10) and pT2-pT4 (n = 4). The boxes represent means ± SEM. The results were obtained with Spearman's rank correlation test (p > 0.05).

An evaluation of *NDRG2 *mRNA expression level, depending on tumour stage in TNM classification was performed for two groups: the first group included pT1 cases (Group pT1, n = 12) and the second one included (because of the small number) combined cases of pT2, pT3 and pT4 (Group pT2-pT4, n = 4) (Figure 3). A comparison of the mean values of *NDRG2 *mRNA expression level between those two groups did not reveal any statistically significant differences (p > 0.05).

An evaluation of the *NDRG2 *mRNA expression level, depending on patient age, was conducted in two groups: in the group of PTC cases in both sexes (primary tumour) at the age till 45 (n = 6) and in the group of cases at the age above 45 (n = 10). The recorded mean values of *NDRG2 *mRNA expression level did not differ in any statistically significant way between those groups (p > 0.05). The calculated correlation coefficient did not show any significant relationship between the studied parameters for the age (Rs = 0.0309; p > 0.1).

The mean values of *NDRG2 *mRNA expression level, recorded in the group of women (n = 12) and in the group of men (n = 4), did not show any statistically significant differences between those groups, either (p > 0.1).

## Discussion

Papillary thyroid carcinoma, despite usually good prognosis and low mortality rates, relatively frequently gives metastases to regional lymph nodes, while, more rarely, distant metastases (mainly to the lungs and bones). PTC spreads mainly via lymphatic vessels. Metastases to regional lymph nodes are observed in more than 50% of operated patients [15]. The most important prognostic factors in PTC include: age, sex, tumour size, encapsulation, infiltration of perithyroid tissues, metastases and postoperative thyroglobulin (Tg) levels [16,17].

In the reported study, the *NDRG2 *mRNA expression levels were evaluated in PTC and in its metastases to regional lymph nodes. In the study, we analysed postoperative tissue material, from primary carcinoma (tumour) and from macroscopically changed lymph nodes, collected from 16 patients of both sexes. Histopathological analysis confirmed PTC presence in tumour tissue and - in 8 cases - metastases of the carcinoma to regional lymph nodes were identified.

Our present results have demonstrated a significantly reduced *NDRG2 *mRNA expression levels in cells of PTC (p < 0.0001). The recorded decrease of the *NDRG2 *mRNA expression level in primary carcinoma (tumour) tissue vs. that in macroscopically unchanged thyroid tissue, is in compliance with the results obtained by Zhao et al. [13]. Those authors have been - till now - the first who have published the results of studies on *NDRG2 *mRNA expression level in PTC. In the study by those authors, decreased *NDRG2 *gene expression levels were found in all the studied cases of PTC (n = 53) [13].

The results of our study confirm also the reports of other authors, analysing the *NDRG2 *gene expression level in various neoplasms [5,6,8-10]. Deng et al. [5] analysed *NDRG2 *gene mRNA levels in cells of glioma and in normal brain tissues. The results of these authors revealed decreased *NDRG2 *expression in approximately 60% of gliomas vs. the expression levels in normal brain tissue. Lusis et al. [8] noted that the expression of *NDRG2 *in glioma was reduced at transcript and protein level. In studies of Norwegian authors, it was demonstrated that mRNA level of *NDRG2 *gene was significantly lower in colon cancer tissue than in normal tissues [10]. Also studies of *NDRG2 *gene expression in gastric carcinoma revealed decreased expression levels of the gene in some of the patients [18]. Moreover, the authors suggested that studies of *NDRG2 *gene expression could be used as a prognostic factor for the survival rate of patients with gastric carcinoma. In the group with decreased expression of *NDRG2 *gene, the survival rate of patients was significantly lower [18].

Other groups of researchers also described decreased expression of *NDRG2 *gene in tissues from various malignant neoplasms, e.g., in breast cancer [19], liver carcinoma [20], clear cell renal carcinoma [21] and skin carcinoma [22].

The process of neoplastic cell metastasis formation includes an intricate complex of molecular and biochemical events ("metastatic cascade"), including: the invasion of primary tumour cells to surrounding tissues, angioinvasion, migration, reimplantation and proliferation of cells. One of the groups of the genes, which participate in these processes, are suppressor genes in the mechanism of metastasis formation, e.g., *nm23, Kai1, KISS1 *[23]. During recent years, also *NDRG2 *gene has been added to the group [7].

In our studies, we found no differences between *NDRG2 *mRNA expression level in primary PTC and mRNA expressions levels in metastases of the carcinoma to regional lymph nodes. However, a positive correlation was noted between *NDRG2 *mRNA expression levels in primary PTC cells and the expression levels of *NDRG2 *mRNA in metastases of the carcinoma to lymph nodes, what means that the decrease of the *NDRG2 *mRNA expression level in the primary PTC was accompanied by decreased mRNA expression levels of *NDRG2 *gene in metastases of the carcinoma to lymph nodes. The results of our study are similar to the results obtained by Zhao et al. [13].

In the present study, we have not defined the role of *NDRG2 *in PTC metastases to lymph nodes; that problem requires further analyses in subsequent studies performed on higher numbers of cases.

In our studies, the prevailing number of cases with PTC (n = 12) was qualified to pT1 group in TNM classification. The other, individual cases of PTC, qualified to pT2, pT3 and pT4, were included into other separate group (n = 4).

A comparison of *NDRG2 *gene expression in the two studied groups of patients with various degree of tumour progression, i.e., in Group pT1 and in Group pT2-pT4, did no show any statistically significant difference. Interestingly, no correlation was documented in the study of Zhao et al. between the expression of *NDRG2 *gene and thyroid tumour size [13].

We also evaluated the correlation between the expression of *NDRG2 *gene and patient's age. We have divided the study group into two subgroups: the age till 45 (n = 6) and above 45 (n = 10). However, an analysis of the correlation between the expression of *NDRG2 *mRNA and the age of examined patients did not show any statistically significant differences. In other reports on different types of neoplasms, including PTC, no correlation was shown between the expression of *NDRG2 *gene and patients' age [10,22]. Moreover, no correlation between the expression of *NDRG2 *and patient's sex, was demonstrated in our study.

## Conclusions

In conclusion, the results of our study demonstrated a decreased level of *NDRG2 *gene expression in PTC vs. macroscopically unchanged thyroid tissue, what may indicate a potential role of *NDRG2 *gene in the development and progression of this neoplasm. No correlations were found between the expression of *NDRG2 *gene and patient's sex or age or the degree in tumour staging (TNM) or its metastases. The evident lack of satisfactory literature data, regarding the studies of *NDRG2 *gene expression in PTC is an encouragement to undertake further studies on a larger groups of patients, which may enable to evaluate the potential of *NDRG2 *gene as a biological marker in PTC.

## Competing interests

The authors declare that they have no competing interests.

## Authors' contributions

AM designed and coordinated the study, carried out the molecular genetic studies and drafted the manuscript. JL participated in performing molecular studies. TF participated in coordination of the study. LP participated in the design of the study. AZ participated in coordination of the study. EG participated in performing molecular studies. AL, the senior author, conceived of the study and wrote the manuscript. All authors have read and approved the final manuscript.
